# Characterizations and Significantly Enhanced Dielectric Properties of PVDF Polymer Nanocomposites by Incorporating Gold Nanoparticles Deposited on BaTiO_3_ Nanoparticles

**DOI:** 10.3390/polym13234144

**Published:** 2021-11-27

**Authors:** Kaniknun Sreejivungsa, Nutthakritta Phromviyo, Ekaphan Swatsitang, Prasit Thongbai

**Affiliations:** 1 Institute of Nanomaterials Research and Innovation for Energy (IN-RIE), NANOTEC-KKU RNN on Nanomaterials Research and Innovation for Energy, Khon Kaen University, Khon Kaen 40002, Thailand; kaniknun_s@kkumail.com (K.S.); ekaphan@kku.ac.th (E.S.); 2 Giant Dielectric and Computational Design Research Group (GD–CDR), Department of Physics, Faculty of Science, Khon Kaen University, Khon Kaen 40002, Thailand; nutthaphrom@gmail.com

**Keywords:** BaTiO_3_, Turkevich method, zeta potential, DSC, polymer nanocomposites

## Abstract

Poly(vinylidene fluoride) (PVDF) nanocomposites were fabricated by incorporating BaTiO_3_ nanoparticles (particle size of ~100 nm, *n*BT), which were deposited by Au nanoparticles (*n*Au) with an average particle size of 17.8 ± 4.0 nm using a modified Turkevich method. Systematic characterizations on the synthesized *n*Au-*n*BT hybrid nanoparticles and *n*Au-*n*BT/PVDF nanocomposites with different contents of a filler were performed. The formation of *n*Au-*n*BT hybrid nanoparticles was confirmed with the calculated *n*Au:*n*BT ratio of 0.5:99.5 wt.%. The homogeneous dispersion of *n*Au and *n*BT in the PVDF polymer was obtained due to the interaction between the negative surface charge of the *n*Au-*n*BT filler (compared to that of the *n*BT) and polar *β*-PVDF phase, which was confirmed by the zeta potential measurement and Fourier-transform infrared spectroscopy, respectively. A significantly increased dielectric permittivity (ε′ ~ 120 at 10^3^ Hz) with a slight temperature-dependent of <±15% ranging from −20 to 140 °C was obtained. Notably, a low loss tangent (tanδ < 0.08) was obtained even at a high temperature of 140 °C. Therefore, incorporating a PVDF polymer with *n*Au-*n*BT hybrid nanoparticles is an attractive method to improve the dielectric properties of a PVDF polymer for dielectrics applications.

## 1. Introduction

Recently, a new method for improving the dielectric and electrical properties of polymer composites, especially for poly(vinylidene fluoride) (PVDF)-based composites, has widely been studied to increase the performance of dielectric polymers [1,2,3,4,5]. Polymer nanocomposites have been focused on due to a large surface area of the nano-filler used, resulting in significantly improved electrical, dielectric, and mechanical properties [4,6,7,8,9,10,11]. To develop dielectric capacitors in embedded devices, polymer composites with low loss tangent (tanδ) and high dielectric permittivity (ε′) are required [1].

The basic method to enhance the dielectric response is to fill high-ε′ ceramic fillers, such as ***A***Cu_3_Ti_4_O_12_ (***A*** = Ca, Na_0.5_Bi_0.5_, Na_0.5_Y_0.5_, and Na_0.33_Ca_0.33_Bi_0.33_) [12,13,14,15], TiO_2_-nanorod [16], ***B***FeO_3_ (***B*** = Bi and La) [17,18], BaTiO_3_ (BT) [1,19], (La, Nb) codoped TiO_2_ [11], and Ba(Fe_0.5_Nb_0.5_)O_3_ [10]) into the polymer matrix. Thus, the giant dielectric oxides, such as TiO_2_-based, La_2-x_Sr_x_NiO_4_, and K_1.53_(Cu_0.76_Ti_7.24_)O_16_ ceramic particles [16,20,21], may be the interesting filler for use to enhance the dielectric response of polymer composites. Unfortunately, the tanδ values of the ceramic/polymer composites are still too large (>0.1) for applications when the volume fraction (*f*_filler_) of a filler is 0.5 [10,14,15,16,19], which significantly deteriorate the electrical breakdown strength of polymer composites.

Another method to enhance the dielectric response is to incorporate conductive nanoparticles, such as Ag [22,23], Al [24], and Ni [25], into the polymer matrix at volumes close to the percolation threshold. The dielectric response is usually dependent on the morphologies and conductivity of conductive fillers [4]. However, the significantly increased ε′ value of the conductor/polymer composites is accompanied by the enormous value of the conductivity and tanδ near the percolation threshold, which is unsuitable for the applications of the polymer composites. It is worth noting that gold nanoparticle (*n*Au) is attractive because of its high electrical conductivity and for being nontoxic. Nevertheless, a designed route for obtaining polymer composites with large ε′ and suppressed tanδ must be performed.

The alternative route to obtain high ε′ with a low tanδ value of a polymer composite is to fill it with a hybrid filler, consisting of ceramic particles with high-ε′ as the primary filler and metal nanoparticle as a minor fille. The discrete growth of metal nanoparticles on the surface of ceramic particles is usually designed, such as Ag-deposited BT nanoparticle (*n*BT) [26,27] and Ag-deposited CaCu_3_Ti_4_O_12_ [28,29], to prevent the continuous contract of the metal nanoparticles, giving rise to the formation of conducting network. Most recently, we found that the dielectric properties of the PVDF polymer composites can be improved by incorporating hybrid particles of Au-deposited BiFeO_3_, Na_0.5_Y_0.5_Cu_3_Ti_4_O_12_, and TiO_2_-nanorod [30,31,32]. Although the *n*Au-*n*BT/PVDF polymer composite is one of the most interesting three-phase composites [33], the systematic characterizations of this composite system have never been reported. Furthermore, the dielectric properties as a function of temperature have also never been reported. The variation of the dielectric properties with temperature is one of the essential data for determining material to use in practical applications.

Thus, in this work, the method to improve the dielectric properties of the PVDF polymer with suppressing tanδ over a wide temperature range was proposed. The *n*Au-*n*BT hybrid nanoparticle with a low *n*Au:*n*BT ratio of 0.5:99.5 was fabricated as a filler in the PVDF matrix. The *n*Au-*n*BT and *n*Au-*n*BT/PVDF nanocomposites were systematically characterized. The dielectric properties were studied and described to evaluate whether such materials are suitable for dielectric capacitor applications.

## 2. Experimental Details

### 2.1. Preparation of nAu-nBT Hybrid Particles

A modified Turkevich method was used to prepare the *n*Au-*n*BT hybrid particles [34], which is a method to reduce Au ions antecedent with sodium citrate in deionized (DI) water. First, 1.4 g of *n*BT powder with an average particle size of ~100 nm (Sigma–Aldrich Co., (St. Louis, MO, USA)) was dispersed in DI water (50 mL) in a beaker to form a white solution at room temperature. Second, the 500 µL of HAuCl_4_.3H_2_O (0.65 mM) (Sigma–Aldrich Co., Louis, MO, USA) solution was added into the homogeneous dispersion of *n*BT particles in the beaker above. Then, the suspension of *n*BT in the HAuCl_4_.3H_2_O solution was heated to ~100 °C under constant stirring. Next, the 4 mL of C_6_H_5_Na_3_O_7_·2H_2_O (Sigma–Aldrich Co, Hamburg, Germany) (38.8 mM) solution was dissolved into the above beaker under constant stirring. Usually, the reaction is complete when the color of the solution changes from white to red color. Then, the red color solution was slowly cooled to 25 °C. After that, the red color solution was centrifuged and washed 3–5 times by DI water. Finally, to achieve the *n*Au-*n*BT hybrid particles, the solution was freeze-dried [33].

### 2.2. Preparation of nAu-nBT/PVDF Nanocomposites

We calculated the volume fractions of the PVDF matrix and filler using the equation,

(1) $$\%\left(nAu-nBT\right)\;by\;weight=\frac{V_{nAu-nBT}\rho_{nAu-nBT}}{V_{nAu-nBT}\rho_{nAu-nBT}+\left(1-V_{PVDF}\right)\rho_{PVDF}}\times 100,$$

where $V_{nAu-nBT}$ is the percentage of *n*Au-*n*BT by volume, $\rho_{nAu-nBT}$ is the theoretical density of *n*Au-*n*BT, $\rho_{PVDF}$ is the density of the PVDF polymer (1.74 g/cm^3^). The $\rho_{nAu-nBT}$ was calculated from the equation,

(2) $$\rho_{nAu-nBT}=\rho_{nBT}V_{nBT}+\rho_{nAu}V_{nAu},$$

where $\rho_{nBT}$ and $\rho_{nAu}$ are the *n*BT (6.08 g/cm^3^) and *n*Au (19.3 g/cm^3^), respectively. $V_{nBT}$ and $V_{nAu}$ are the volume fractions of the *n*BT and *n*Au, respectively. We showed that the percentage ratio of *n*Au:*n*BT was obtained to be 0.5:99.5 wt%. By using Equations (1) and (2), the $\rho_{nAu-nBT}$ was calculated and found to be 6.1 g/cm^3^. Thus, the nanocomposites with different volume fractions of *n*Au-*n*BT hybrid particles can be designed in the experimental using Equation (1). The PVDF polymer nanocomposites filled with *n*Au-*n*BT hybrid particles were fabricated. First, PVDF powder (M_w_ ~ 534,000, Sigma–Aldrich, Saint-Quentin-Fallavier, France) was thoroughly mixed with the *n*Au-*n*BT powder in different volume fractions (*f_n_*_Au-*n*BT_) by a wet ball-milling method for 3 h in ethanol using ZrO_2_ balls as a grinding media. Second, ZrO_2_ balls were separated from the mixed PVDF and *n*Au-*n*BT powders. Then, the ethanol was evaporated from the mixture by heating at 80 °C for 24 h. After that, the mixed powder was molded by hot pressing at 200 °C under 10 MPa for 0.5 h. Finally, the *n*Au-*n*BT/PVDF nanocomposite disks with a thickness of ~0.6–1 mm and a diameter of ~12 mm were achieved. Note that the *n*BT/PVDF nanocomposites were also fabricated using the same method to show the effect of the addition *n*Au particles.

### 2.3. Characterization Techniques and Dielectric Measurement

The phase compositions of the *n*Au-*n*BT powders and *n*Au-*n*BT/PVDF composites were investigated using X-ray diffraction (XRD; PANalytical, EMPYREAN, Shanghai, China). The morphologies of the *n*BT and *n*Au-*n*BT particles were revealed using transmission electron microscopy (TEM; FEI Tecnai G2 20, Eindhoven, The Netherlands). The *n*Au-*n*BT powder was also characterized by UV-vis absorption spectroscopy (Shimadzu, UV-1800, Beachwood, OH, USA). The stability properties and the electrostatic surface charge of *n*BT, *n*Au, and *n*Au-*n*BT were measured using Zetasizer (ZS-90 Malvern Instruments, Manchester, UK). Field-emission scanning electron microscopy (FE-SEM, FEI Helios Nanolab G3 CX) was used to show the dispersion of the *n*Au-*n*BT particles, while energy dispersive X-ray spectroscopy (EDS) was used to determine the elemental composition of the nanocomposites. FE-SEM-EDS mapping images were also corrected. Prior to characterizing the morphologies, the nanocomposites were immersed in liquid N_2_ for 5 min. After that, the nanocomposites were fractured. Then, the cross-sections of the nanocomposites were coated by Au sputtering for 2 min. The phases of the PVDF polymer matrix were characterized using Fourier transform infrared spectroscopy (FTIR; Bruker, TENSOR27, Bremen, Germany). The thermal behavior of *n*Au-*n*BT/PVDF nanocomposite was characterized by differential scanning calorimetry (DSC, PerkinElmer, 8000 Advanced Double-Furnace, Solingen, Germany).

Before dielectric measurements, the disk samples were painted on both sides with silver paste and then dried at 100 °C for 30 min. The dielectric properties of the *n*Au-*n*BT/PVDF nanocomposites were investigated using an impedance analyzer (KEYSIGHT, E4990A, Santa Rosa, CA, USA) under an AC oscillation voltage of 500 mV over a frequency range of 10^2^–10^6^ Hz and temperature range of −60 to 140 °C.

## 3. Results and Discussion

**Figure 1:**
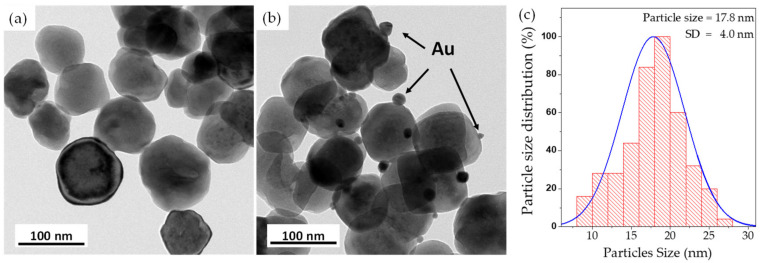
TEM images of the surface morphologies of (**a**) *n*BT and (**b**) *n*Au-*n*BT particles. (**c**) Size distribution of *n*Au nanoparticles deposited on *n*BT.

The TEM images of the morphologies of *n*BT particles and *n*Au-*n*BT hybrid particles are revealed in Figure 1a,b, respectively. The *n*BT particles are nearly spherical in shape with a particle size of ~100 nm. The *n*Au particles have a spherical shape and are randomly deposited on the surface of *n*BT particles. As shown in Figure 1c, the size distribution of *n*Au is narrow with an average particle size of ~17.8 ± 4.0 nm, which is comparable to the particle size of *n*Au prepared by the Turkevich method reported in the literature [33,34].

**Figure 2:**
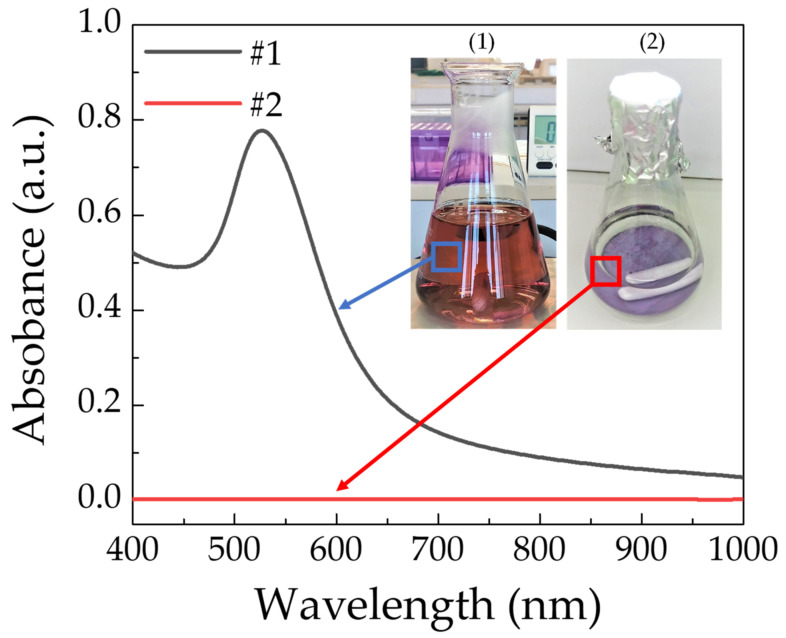
UV-vis spectra of HAuCl_4_.3H_2_O solution (1) and separated transparency solution after HAuCl_4_.3H_2_O solution (2) was centrifuged; insets (1) and (2) show the photo images corresponding to the UV-vis spectra #1 and #2.

To demonstrate the complete deposition of *n*Au particles on the surface of *n*BT, UV-vis spectroscopy was used to examine the existence of *n*Au particles. As shown in Figure 2, the noticeable absorbance peak at the wavelength of ~520 nm was observed in the UV-vis spectrum of the HAuCl_4_.3H_2_O solution (red color solution in the inset (1)), indicating the formation of *n*Au particles in this process [35]. This result is similar to those reported in the literature [35,36]. After the above solution was centrifuged to precipitate the *n*Au-*n*BT powder, the separated transparency solution (inset (2)) was further characterized by UV-vis spectroscopy. The UV-vis spectrum showed no absorbance peak, confirming that there was no residual *n*Au in the separated transparency solution. This result confirmed that most all of the *n*Au can be reacted completely and decorated on the surface of *n*BT. This result was consistent with that observed in the TEM image, as shown in Figure 1b. According to the designed starting raw materials used for fabricating the *n*Au-*n*BT hybrid particles, the percentage ratio of the *n*Au:*n*BT in the starting materials used was 0.5:99.5 wt%. Thus, the ratio of the *n*Au:*n*BT in the obtained *n*Au-*n*BT hybrid particles should be ≈5:99.5 wt%.

**Figure 3:**
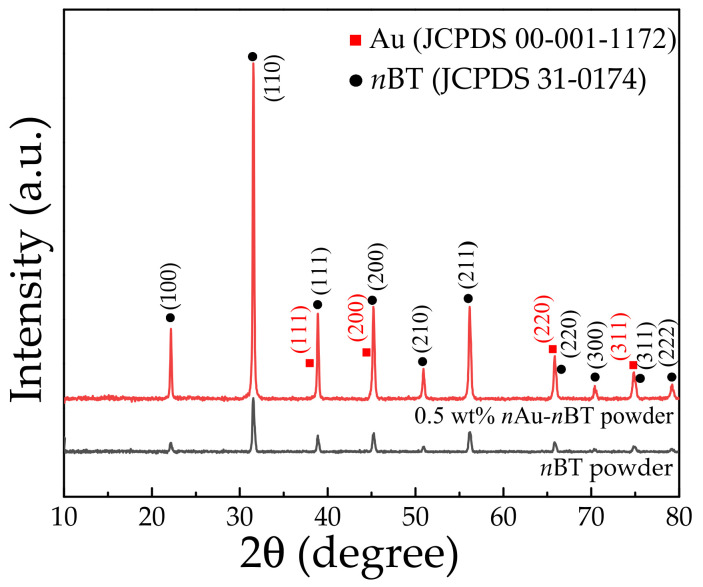
XRD patterns of *n*BT powder and *n*Au-*n*BT hybrid powder.

The XRD patterns in the 2θ range of 10°–70° for the *n*Au-*n*BT and *n*BT powders are displayed in Figure 3. The BT phase was confirmed in both the *n*Au-*n*BT and *n*BT powders. The tetragonal phase structure of BT can usually be detected at room temperature. As shown in the inset of Figure 3, the characteristic peak at 2θ ≈ 45° ((200) plane) of the *n*BT showed a single peak, indicating a cubic phase structure of the *n*BT particles used due to the nanoparticle form [26,37]. The XRD pattern of the *n*Au-*n*BT powder also showed the characteristic peaks corresponding to the (111) and (200) planes of Au (JCPDS 00-001-1172), confirming the presence of *n*Au in the hybrid powder. Although Au is expensive, it was used in minimal quantities compared to that of the silver (Ag)-deposited *n*BT hybrid particles [26,27,38] or Ni-Na_0.33_Ca_0.33_Bi_0.33_Cu_3_Ti_4_O_12_ [39].

**Figure 4:**
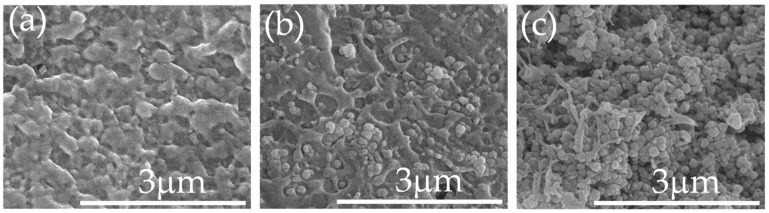
FE-SEM of fractured surface for *n*Au-*n*BT/PVDF nanocomposites with *f_n_*_Au-*n*BT_ = (**a**), 0 (PVDF), (**b**), 0.1 and (**c**) 0.5.

The obtained *n*Au-*n*BT hybrid particles were incorporated into the PVDF matrix with different contents for fabricating the *n*Au-*n*BT/PVDF nanocomposites. The FE-SEM images of the morphologies of fractured surfaces of the PVDF polymer and *n*Au-*n*BT/PVDF nanocomposites with *f_n_*_Au-*n*BT_ = 0.1 and 0.5 are shown in Figure 4. The continuous phase of the PVDF polymer was observed, as shown in Figure 4a. The *n*Au-*n*BT particles were randomly distributed throughout the PVDF matrix with a small degree of agglomeration, as shown in Figure 4b. The morphology of the nanocomposite with *f_n_*_Au-*n*BT_ = 0.5 is shown in Figure 4c. The continuous phase of the PVDF matrix decreased. A small number of pores were observed due to the increased *f_n_*_Au-*n*BT_.

**Figure 5:**
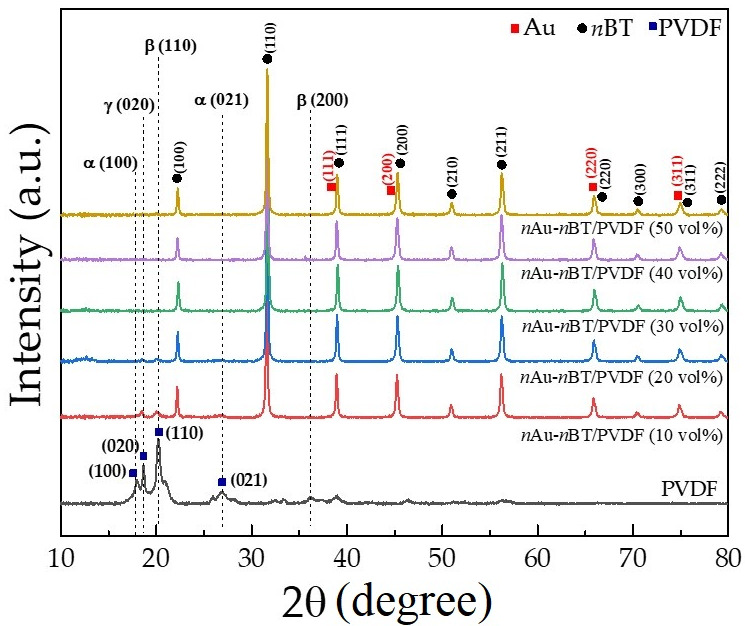
XRD patterns of PVDF and *n*Au-*n*BT/PVDF nanocomposites with various contents of *n*Au-*n*BT (*f_n_*_Au-*n*BT_ = 0.1–0.5).

To confirm the phase compositions, the pure PVDF polymer and *n*Au-*n*BT/PVDF nanocomposites with various contents of *n*Au-*n*BT hybrid (10–50 vol%) were characterized using the XRD technique. As indicated in Figure 5, the non-polar α-phase and the polar *β*- and *γ*-phases were detected in the XRD pattern of the PVDF polymer. The diffraction peaks at 2θ ≈ 18°and 26.7° can be assigned as the α-phase, corresponding to the (100) and (021) planes, respectively. The *β*-phase peak was indexed at 2θ ≈ 20.4° and 36.6°, corresponding to the (110) and (200) planes, respectively. The γ-phase peak was detected at 2θ ≈ 18.6°, corresponding to the (020) plane [3,40,41,42]. For the *n*Au-*n*BT/PVDF nanocomposites with low *f_n_*_Au-*n*BT_, the intensity of these PVDF-phases was very low due to the high crystallinity of the filler. Furthermore, all PVDF-phases cannot be observed in the *n*Au-*n*BT/PVDF nanocomposites with high *f_n_*_Au-*n*BT_. The phases of a PVDF polymer in the composites can be formed in several phases, depending on to preparation method and types of fillers [2,3].

**Figure 6:**
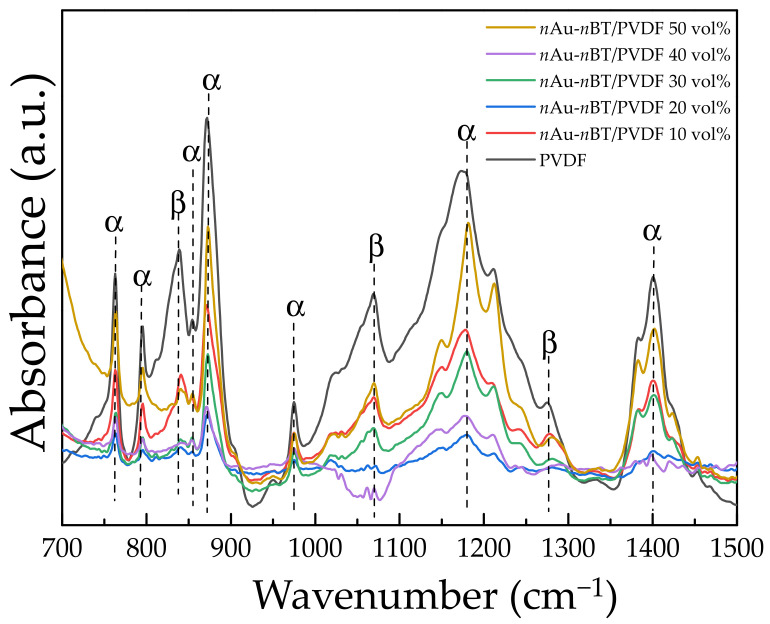
FTIR spectra of *n*Au-*n*BT/PVDF nanocomposites with various contents of *n*Au-*n*BT (*f_n_*_Au-*n*BT_ = 0.1–0.5).

The *n*Au-*n*BT/PVDF nanocomposites were further characterized using the FTIR technique to identify the phases of the PVDF polymer matrix. Figure 6 shows the FTIR spectra of the PVDF and *n*Au-*n*BT/PVDF nanocomposites. The FTIR peaks for the α-phase were observed at the wavenumbers of 766, 791, 873, 1179, and 1401 cm^−1^ [40,41]. The peak at 840, 1072, and 1275 cm^−1^ confirmed the presence of the β-phase of PVDF polymers [41]. It is worth noting that the characteristic peaks of the β- and γ-phases around 840–841 cm^−1^ were very close. The absorption values at 766 (Aα) and 840 cm^−1^ (A_β_) for the α and β phases, respectively, were used to calculate the β-phase content (F(β)) [41], as the following equation.

(3) $$F\left(\beta \right)=\frac{A_{\beta }}{\left(\frac{K_{\beta }}{K_{\alpha }}\right)A_{\alpha }+A_{\beta }},$$

where K_α_ and K_β_ are the absorption coefficients of the α and β phases, the values of which were 6.1 × 10^4^ and 7.7 × 10^4^ cm^2^·mol^−1^, respectively [41]. The F(β) values of the *n*Au-*n*BT/PVDF nanocomposites with *f_n_*_Au-*n*BT_ = 0.1, 0.2, 0.3, 0.4, and 0.5 were 56, 53, 45, 43, 41, and 39%, respectively. The polar β-phase in the nanocomposites was dependent on the relative volume fraction.

The zeta potential measurements of the *n*Au, *n*BT, and *n*Au-*n*BT hybrids were performed by dispersing these particles in DI water to examine the electrostatic surface charge. The zeta potential of *n*BT cannot be measured because the dispersion of *n*BT was not stable in DI water, which precipitated in a few minutes. Nevertheless, the average zeta potential values of the *n*Au and *n*Au-*n*BT were obtained to be −23.33 ± 4.05 mV and −16.87 ± 0.65 mV, respectively. The *n*BT and *n*Au-*n*BT were further dispersed in dimethyl sulfoxide (DMSO) solution for measuring the zeta potential values. The average zeta potential values of the *n*BT and *n*Au-*n*BT were obtained to be 6.26 ± 1.20 mV and −6.62 ± 0.16 mV, respectively. The average positive-electrostatic surface charge of the *n*BT was changed to a negative charge by depositing with *n*Au particles. This result also confirmed that the *n*Au can decorate on the surface.

**Figure 7:**
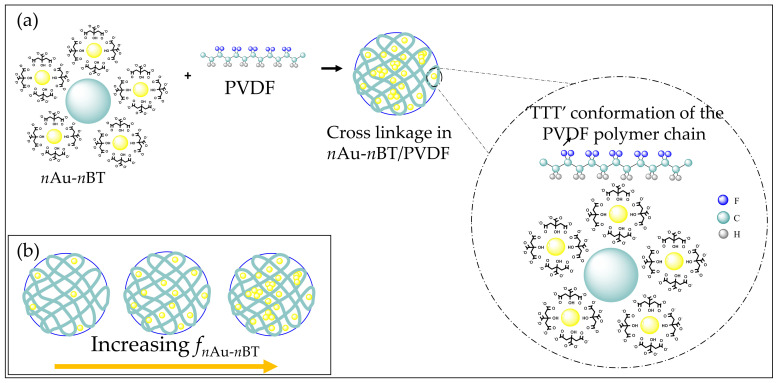
(**a**) Interaction between –CH_2_ dipole of PVDF polymer and negatively surface charge of *n*Au-*n*BT hybrid particles. (**b**) Schematic of loading of different concentrations of *n*Au-*n*BT filler into PVDF polymer chain with an increasing vol% of *n*Au-*n*BT.

As demonstrated in Figure 7a, for a low *f_n_*_Au-*n*BT_, the *n*Au-*n*BT hybrid particles with a negative surface charge interacted with the positive charge (–CH_2_ dipole) of the *β-*PVDF polymer matrix, resulting in good compatibility between the PVDF polymer and a hybrid filler (Figure 4b) due to a large F(β). As demonstrated in Figure 7b, for a high *f_n_*_Au-*n*BT_, the significantly decreased F(β) resulted in the agglomeration of *n*Au-*n*BT hybrid particles (Figure 4c). This is because the total negative charge of the hybrid particles in the composite is larger than the total positive charges of the *β-*PVDF polymer.

**Figure 8:**
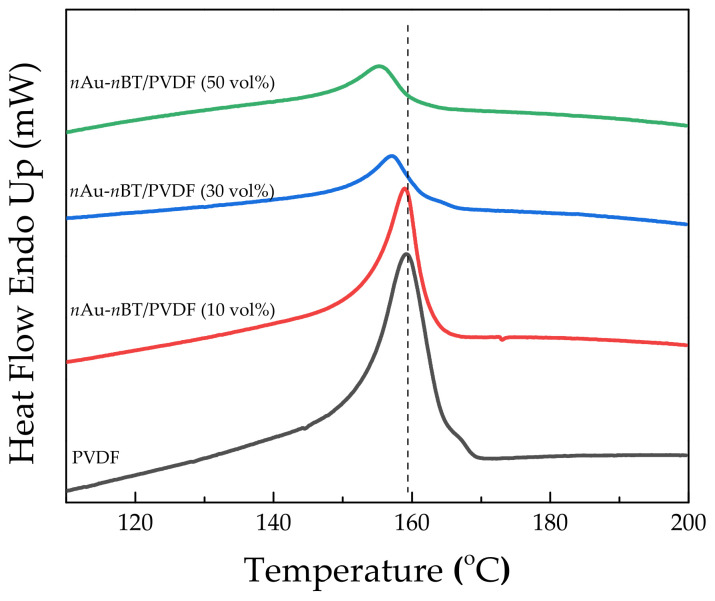
DSC thermograph of PVDF and *n*Au-*n*BT/PVDF nanocomposites with various contents of *n*Au-*n*BT.

Figure 8 shows the DSC curves of the PVDF and *n*Au-*n*BT/PVDF nanocomposites with *f_n_*_Au-*n*BT_ = 0.1, 0.3, and 0.5. The incorporation of *n*Au-*n*BT hybrid particles into the PVDF polymer caused a slight decrease in the melting temperature (*T*_m_), which was similar to that observed in the *n*Au-*n*BT/PVDF composites [32]. As shown in Table 1, the melting enthalpy (*ΔH_m_*) of the nanocomposites decreased significantly as the *n*Au-*n*BT increased. The *X_c_* of polymer nanocomposite was calculated using the following equation (4).

(4) $$X_{c}=\frac{∆H_{m}}{∆H_{m100\%}}\times 100\%,$$

where Δ*H_m_* and Δ*H_m_*_100%_ are the melting enthalpies of crystallization of the nanocomposites and the melting enthalpy of fusion of pure crystallization PVDF (104.7 J g^−1^), respectively [32]. The *X_c_* of the PVDF polymer matrix reduced from 39.53% to 5.58% for the nanocomposite with *f_n_*_Au-*n*BT_ = 0.5, which may be attributed to the inhibited polymer chain movement by the hybrid filler.

**Figure 9:**
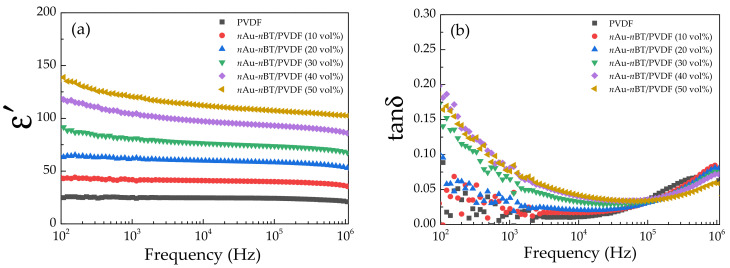
(**a**) Dielectric permittivity (ε′) and (**b**) dielectric loss tangent (tanδ) at 25 °C (10^2^–10^6^ Hz) of PVDF and *n*Au-*n*BT/PVDF nanocomposites with different contents of *n*Au-*n*BT.

Figure 9 displays the dielectric properties at 25 **°**C in the frequency range of 10^2^–10^6^ Hz for the PVDF and *n*Au-*n*BT/PVDF nanocomposites. As shown in Figure 9a, the dielectric response in the *n*Au-*n*BT/PVDF nanocomposites continuously increased as the filler loading increased. Furthermore, the ε′ in the range of 10^3^–10^6^ Hz was almost independent of the frequency. However, the ε′ was in the range of 10^2^–10^3^ Hz and slightly decreased with increasing frequency. This result is usually due to interfacial polarization, especially at the interface between the nanocomposite sample and electrode [43]. The slight reduction in ε′ at the frequency of 10^6^ Hz can be explained by the dielectric relaxation of dipolar polarization due to the orientation of C-F in the PVDF matrix [44]. The *n*Au-*n*BT/PVDF nanocomposites with *f_n_*_Au-*n*BT_ = 0.1, 0.2, 0.3, 0.4, and 0.5 exhibited the largely increased ε′ values of ~41.79, 61.93, 80.73, 103.88, and 120.35, respectively (at 10^3^ Hz). It is important to note that the ε′ value of the *n*Au-*n*BT/PVDF nanocomposite with *f_n_*_Au-*n*BT_ = 0.4 was much larger than that of the two-phase *n*BT/PVDF nanocomposite with *f_n_*_BT_ = 0.4 (ε′ ~ 66.1) [19]. Thus, the significantly increased dielectric response in the *n*Au-*n*BT/PVDF nanocomposites should be correlated to the presence of *n*Au particles. For the two-phase *n*BT/PVDF nanocomposites, the primary cause of the enhanced dielectric response is due to the high ε′ value of the *n*BT particles compared to that of the PVDF polymer matrix [19], while the greatly enhanced dielectric response is due to the interfacial polarization which can only be dominant in a low-frequency range [37]. For the three-phase *n*Au-*n*BT/PVDF nanocomposites, the significantly increased dielectric response over the measured frequency range is likely due to the Maxwell–Wagner–Sillars (MWS) polarization at the *n*BT-*n*Au and PVDF-*n*Au interfaces [45]. The MWS effect was related to the trapped free charges at the discontinuous surfaces between the insulating and conducting phases. Hence, macroscopic dipoles could be created under an applied electric field, giving rise to the enhancement of the dielectric response in the three-phase nanocomposites. Furthermore, good compatibility between the PVDF polymer matrix and the hybrid filler particles is another cause of the observed increased dielectric response.

**Figure 10:**
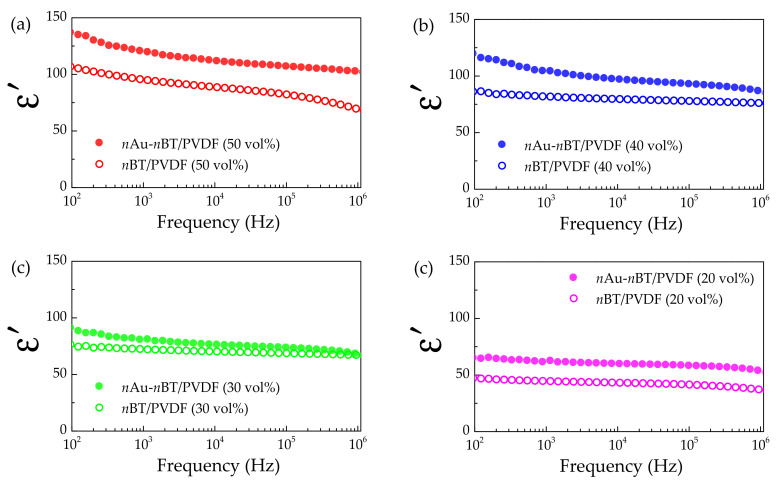
Comparison of dielectric permittivity (ε′) at 25 °C of *n*BT/PVDF and *n*Au-*n*BT/PVDF nanocomposites with different volume fractions: (**a**) 0.5, (**b**) 0.4, (**c**) 0.3, and (**d**) 0.2.

The tanδ values of the *n*Au-*n*BT/PVDF nanocomposites are shown in Figure 9b. A low-frequency tanδ value tended to increase with increasing *f_n_*_Au-*n*BT_. The increased tanδ in a low-frequency range is attributed to the interfacial polarization associated with the DC conduction at the interface between the nanocomposite sample and electrodes, corresponding to the observed decrease in the low-frequency ε′. At 10^3^ Hz, the *n*Au-*n*BT/PVDF nanocomposite with *f_n_*_Au-*n*BT_ = 0.5 was 0.076. It can be explained that the suppressed tanδ has resulted from the modified surface of the *n*BT particles. The surface of *n*BT was still an insulating surface, in which the long-range motion of free charge carriers was inhibited by preventing the direct contact between *n*Au particles [26,30,31,32,38]. The sharp increase in tanδ in a high-frequency range was correlated to the decreased ε′, which originated by the α_a_ relaxation due to the glass transition of the pure PVDF polymer [46]. Notably, the dielectric properties of the *n*Au-*n*BT/PVDF nanocomposite with *f_n_*_Au-*n*BT_ = 0.5 were greatly be improved with increased ε′ of ~120 and retained low tanδ. The improved dielectric properties of the *n*Au-*n*BT/PVDF nanocomposites were comparable to those of three-phase polymer nanocomposites [26,30,31,32]. The effect of *n*Au particles on the dielectric response of the *n*Au-*n*BT/PVDF nanocomposites with different *f_n_*_Au-*n*BT_ is demonstrated in Figure 10a,d. The ε′ values of the *n*Au-*n*BT/PVDF nanocomposites were compared to those values of the *n*BT/PVDF nanocomposites. As clearly seen, the ε′ values of the *n*BT/PVDF nanocomposites increased by incorporating a small number of *n*Au particles. These results indicated that the addition of *n*Au particles produced interfacial polarization, giving rise to the enhanced ε′ value.

**Figure 11:**
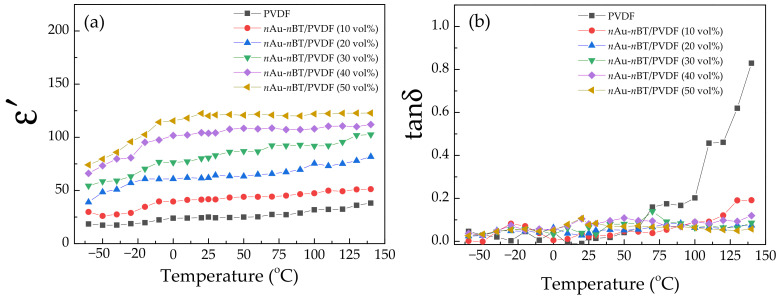
(**a**) Dielectric permittivity (ε′) and (**b**) dielectric loss tangent (tanδ) at 10^3^ Hz (−60–140 °C) of PVDF and *n*Au-*n*BT/PVDF nanocomposites with different contents of *n*Au-*n*BT.

Besides the increased ε′ value with retaining tanδ, the temperature stability of the dielectric properties was also important to study the polymer nanocomposites in practical applications. The dielectric properties at 10^3^ Hz as a function of temperature are shown in Figure 11. As depicted in Figure 11a, the ε′ of the *n*Au-*n*BT/PVDF nanocomposites with *f_n_*_Au-*n*BT_ = 0.4 and 0.5 showed good stability at temperatures ranging from −20 to 140 °C According to the EIA standard [47], the changes in ε′ at 1 kHz (or capacitance change) with a temperature of the *n*Au-*n*BT/PVDF nanocomposites with *f_n_*_Au-*n*BT_ = 0.4 and 0.5 were, respectively, limited to ±7.5% and ±2.2% in the temperature range from +10 °C to +130 °C compared to the ε′ value at 25 °C, which was the operating temperature range for the Z7F and Z7C (+10 °C to +125 °C) capacitors, respectively. The rapid change in ε′ at a low-temperature range resulted from the freezing of the dipole moments [26]. As shown in Figure 11b, the tanδ of the *n*Au-*n*BT/PVDF nanocomposites was slightly dependent on the temperature over the measured range, while the tanδ of the PVDF polymer greatly increased as the temperature increased, which was the α-relaxation [48]. Notably, a low tanδ (<0.08) of the *n*Au-*n*BT/PVDF nanocomposite with *f_n_*_Au-*n*BT_ = 0.5 was achieved even at a high temperature of 140 °C.

## 4. Conclusions

The successful fabrication of *n*Au-*n*BT hybrid particles using a modified Turkevich method was demonstrated. The hybrid particles were used as a filler in the PVDF polymer to fabricate dielectric polymer nanocomposites. Greatly increased ε′ values and low tanδ values were obtained in the *n*Au-*n*BT/PVDF nanocomposites. Furthermore, the dielectric properties of the nanocomposites were stable with temperature over a wide temperature range. The *n*Au-*n*BT hybrid particles can increase the dielectric response of the polymer nanocomposites via the creation of interfacial polarization. The suppressed tanδ was due to a small number of conductive *n*Au particles used, which were discretely grown on the surface of the *n*BT particles. This observation can also cause the improved temperature stability of the dielectric properties of the nanocomposites.

## Figures and Tables

**Table 1:** Melting temperature (*T*_m_), melting enthalpy (Δ*H*_m_), and crystallinity degree (*X*_C_) of *n*Au-*n*BT/PVDF nanocomposites with *f_n_*_Au-*n*BT_ = 0.1, 0.2, and 0.3.

*f_n_*_Au-*n*BT_	*T*_m_ (°C)	Δ*H*_m_ (J g^−1^)	*X*_C_ (%)
0 (PVDF)	159.17	41.39	39.53
0.1	158.94	27.30	26.07
0.3	157.12	11.59	11.07
0.5	155.25	10.03	5.58

## Data Availability

The data presented in this study are available on request from the corresponding author.

## References

[1] Dang Z.-M., Yuan J.-K., Zha J.-W., Zhou T., Li S.-T., Hu G.-H. (2012). Fundamentals, processes and applications of high-permittivity polymer–matrix composites. Prog. Mater. Sci..

[2] Ribeiro C., Costa C.M., Correia D.M., Nunes-Pereira J., Oliveira J., Martins P., Gonçalves R., Cardoso V.F., Lanceros-Méndez S. (2018). Electroactive poly(vinylidene fluoride)-based structures for advanced applications. Nat. Protoc..

[3] Thakur V.K., Gupta R.K. (2016). Recent Progress on Ferroelectric Polymer-Based Nanocomposites for High Energy Density Capacitors: Synthesis, Dielectric Properties, and Future Aspects. Chem. Rev..

[4] Nan C.W., Shen Y., Ma J. (2010). Physical Properties of Composites Near Percolation. Annu. Rev. Mater. Res..

[5] You X., Chen N., Du G. (2018). Constructing three-dimensionally interwoven structures for ceramic/polymer composites to exhibit colossal dielectric constant and high mechanical strength: CaCu_3_Ti_4_O_12_/epoxy as an example. Compos. Part A Appl. Sci. Manuf..

[6] Yang P., Tian K., Ren X., Zhou K. (2019). A comparative study of electrical aging of multiwalled carbon nanotubes and carbon black filled cross-linked polyethylene. Nanocomposites.

[7] Aigbodion V.S. (2021). Explicit microstructure and electrical conductivity of epoxy/carbon nanotube and green silver nanoparticle enhanced hybrid dielectric composites. Nanocomposites.

[8] Singh T., Tiwari S.K., Shukla D.K. (2020). Effects of Al_2_O_3_ nanoparticles volume fractions on microstructural and mechanical characteristics of friction stir welded nanocomposites. Nanocomposites.

[9] Prasad A.S., Wang Y., Li X., Iyer A., Chen W., Brinson L.C., Schadler L.S. (2020). Investigating the effect of surface modification on the dispersion process of polymer nanocomposites. Nanocomposites.

[10] Wang Z., Fang M., Li H., Wen Y., Wang C., Pu Y. (2015). Enhanced dielectric properties in poly(vinylidene fluoride) composites by nanosized Ba(Fe_0.5_Nb_0.5_)O_3_ powders. Compos. Sci. Technol..

[11] Zeng Y., Xiong C., Li J., Huang Z., Du G., Fan Z., Chen N. (2021). Structural, dielectric and mechanical behaviors of (La, Nb) Co-doped TiO_2_/Silicone rubber composites. Ceram. Int..

[12] Kaur S., Singh D.P. (2020). On the structural, dielectric and energy storage behaviour of PVDF-CaCu_3_Ti_4_O_12_ nanocomposite films. Mater. Chem. Phys..

[13] Kum−onsa P., Thongbai P. (2020). Na_1/3_Ca_1/3_Bi_1/3_Cu_3_Ti_4_O_12_/poly(vinylidene fluoride) composites with high dielectric permittivity and low dielectric loss. Mater. Chem. Phys..

[14] Kum-onsa P., Thongbai P. (2020). Improved Dielectric Properties of Poly(vinylidene fluoride) Composites Incorporating Na_1/2_Y_1/2_Cu_3_Ti_4_O_12_ Particles. Mater. Today Commun..

[15] Su Y.-l., Sun C., Zhang W.-Q., Huang H. (2013). Fabrication and dielectric properties of Na_0.5_Bi_0.5_Cu_3_Ti_4_O_12_/poly(vinylidene fluoride) composites. J. Mater. Sci..

[16] Kum-Onsa P., Chanlek N., Thongbai P. (2021). Largely enhanced dielectric properties of TiO_2_-nanorods/poly(vinylidene fluoride) nanocomposites driven by enhanced interfacial areas. Nanocomposites.

[17] Kum P., Thongbai P. (2021). Dielectric properties of poly(vinylidene fluoride)-based nanocomposites containing a LaFeO_3_ nanoparticle filler. J. Mater. Sci. Mater. Electron..

[18] Dash S., Choudhary R.N.P., Goswami M.N. (2017). Enhanced dielectric and ferroelectric properties of PVDF-BiFeO_3_ composites in 0–3 connectivity. J. Alloys Compd..

[19] Silakaew K., Saijingwong W., Meeporn K., Maensiri S., Thongbai P. (2015). Effects of processing methods on dielectric properties of BaTiO_3_/poly(vinylidene fluoride) nanocomposites. Microelectron. Eng..

[20] Gorshkov N., Vikulova M., Gorbunov M., Mikhailova D., Burmistrov I., Kiselev N., Artyukhov D., Gorokhovsky A. (2021). Synthesis of the hollandite-like copper doped potassium titanate high-k ceramics. Ceram. Int..

[21] Meeporn K., Thongbai P. (2020). Flexible La_1.5_Sr_0.5_NiO_4_/Poly(vinylidene fluoride) composites with an ultra high dielectric constant: A comparative study. Compos. Part B Eng..

[22] Huang X., Jiang P., Xie L. (2009). Ferroelectric polymer/silver nanocomposites with high dielectric constant and high thermal conductivity. Appl. Phys. Lett..

[23] Phromviyo N., Chanlek N., Thongbai P., Maensiri S. (2018). Enhanced dielectric permittivity with retaining low loss in poly(vinylidene fluoride) by incorporating with Ag nanoparticles synthesized via hydrothermal method. Appl. Surf. Sci..

[24] Zhou W., Zuo J., Ren W. (2012). Thermal conductivity and dielectric properties of Al/PVDF composites. Compos. Part A Appl. Sci. Manuf..

[25] Wang Z., Zhou W., Dong L., Sui X., Cai H., Zuo J., Chen Q. (2016). Dielectric spectroscopy characterization of relaxation process in Ni/epoxy composites. J. Alloys Compd..

[26] Luo S., Yu S., Sun R., Wong C.-P. (2014). Nano Ag-Deposited BaTiO_3_ Hybrid Particles as Fillers for Polymeric Dielectric Composites: Toward High Dielectric Constant and Suppressed Loss. ACS Appl. Mater. Interfaces.

[27] Silakaew K., Chanlek N., Manyam J., Thongbai P. (2021). Highly enhanced frequency- and temperature-stability permittivity of three-phase poly(vinylidene-fluoride) nanocomposites with retaining low loss tangent and high permittivity. Results Phys..

[28] Ghosh B., Tamayo Calderón R.M., Espinoza-González R., Hevia S.A. (2017). Enhanced dielectric properties of PVDF/CaCu_3_Ti_4_O_12_:Ag composite films. Mater. Chem. Phys..

[29] Yang Y., Sun H., Yin D., Lu Z., Wei J., Xiong R., Shi J., Wang Z., Liu Z., Lei Q. (2015). High performance of polyimide/CaCu_3_Ti_4_O_12_@Ag hybrid films with enhanced dielectric permittivity and low dielectric loss. J. Mater. Chem. A.

[30] Kum-onsa P., Chanlek N., Manyam J., Thongbai P., Harnchana V., Phromviyo N., Chindaprasirt P. (2021). Gold-Nanoparticle-Deposited TiO_2_ Nanorod/Poly(Vinylidene Fluoride) Composites with Enhanced Dielectric Performance. Polymers.

[31] Kum-onsa P., Chanlek N., Putasaeng B., Thongbai P. (2020). Improvement in dielectric properties of poly(vinylidene fluoride) by incorporation of Au–BiFeO_3_ hybrid nanoparticles. Ceram. Int..

[32] Kum-onsa P., Phromviyo N., Thongbai P. (2020). Suppressing loss tangent with significantly enhanced dielectric permittivity of poly(vinylidene fluoride) by filling with Au–Na_1/2_Y_1/2_Cu_3_Ti_4_O_12_ hybrid particles. RSC Adv..

[33] Phromviyo N., Thongbai P., Maensiri S. (2018). High dielectric permittivity and suppressed loss tangent in PVDF polymer nanocomposites using gold nanoparticle–deposited BaTiO_3_ hybrid particles as fillers. Appl. Surf. Sci..

[34] Turkevich J., Stevenson P.C., Hillier J. (1951). A study of the nucleation and growth processes in the synthesis of colloidal gold. Discuss. Faraday Soc..

[35] Ji X., Song X., Li J., Bai Y., Yang W., Peng X. (2007). Size Control of Gold Nanocrystals in Citrate Reduction: The Third Role of Citrate. J. Am. Chem. Soc..

[36] Tran M., DePenning R., Turner M., Padalkar S. (2016). Effect of citrate ratio and temperature on gold nanoparticle size and morphology. Mater. Res. Express.

[37] Fan B.-H., Zha J.-W., Wang D., Zhao J., Dang Z.-M. (2012). Size-dependent low-frequency dielectric properties in the BaTiO_3_/poly(vinylidene fluoride) nanocomposite films. Appl. Phys. Lett..

[38] Silakaew K., Thongbai P. (2019). Suppressed loss tangent and conductivity in high-permittivity Ag-BaTiO_3_/PVDF nanocomposites by blocking with BaTiO_3_ nanoparticles. Appl. Surf. Sci..

[39] Kum-onsa P., Phromviyo N., Thongbai P. (2020). Na_1/3_Ca_1/3_Bi_1/3_Cu_3_Ti_4_O_12_–Ni@NiO/poly(vinylidene fluoride): Three–phase polymer composites with high dielectric permittivity and low loss tangent. Results Phys..

[40] Cai X., Lei T., Sun D., Lin L. (2017). A critical analysis of the α, β and γ phases in poly(vinylidene fluoride) using FTIR. RSC Adv..

[41] Martins P., Lopes A.C., Lanceros-Mendez S. (2014). Electroactive phases of poly(vinylidene fluoride): Determination, processing and applications. Prog. Polym. Sci..

[42] RP V., Khakhar D.V., Misra A. (2010). Studies on α to β phase transformations in mechanically deformed PVDF films. J. Appl. Polym. Sci..

[43] Arbatti M., Shan X., Cheng Z.Y. (2007). Ceramic–Polymer Composites with High Dielectric Constant. Adv. Mater..

[44] Wang Z., Wang T., Fang M., Wang C., Xiao Y., Pu Y. (2017). Enhancement of dielectric and electrical properties in BFN/Ni/PVDF three-phase composites. Compos. Sci. Technol..

[45] Lin J., Zhang P., Yang W., Xie Z., Liu Y., Lin H., Li X., Lei Q. (2014). Novel potassium sodium niobate/polyimide functional composite films with high dielectric permittivity. Polym. Compos..

[46] Liu S., Xue S., Zhang W., Zhai J., Chen G. (2014). Significantly enhanced dielectric property in PVDF nanocomposites flexible films through a small loading of surface-hydroxylated Ba0.6Sr0.4TiO3nanotubes. J. Mater. Chem. A.

[47] Moulson A.J., Herbert J.M. (2003). Electroceramics: Materials, Properties, Applications.

[48] Lopes A.C., Costa C.M., i Serra R.S., Neves I.C., Ribelles J.L.G., Lanceros-Méndez S. (2013). Dielectric relaxation, ac conductivity and electric modulus in poly(vinylidene fluoride)/NaY zeolite composites. Solid State Ion..

